# Isolation and Characterization of *Enterococcus faecalis* Phage ZXL-01 and Preliminary Investigation of Its Therapeutic Effect on Periapical Periodontitis

**DOI:** 10.3390/cimb47060469

**Published:** 2025-06-18

**Authors:** Hailin Jiang, Xueli Zhao, Chuhan Wang, Hongyan Shi, Jinghua Li, Chunyan Zhao, Honglan Huang

**Affiliations:** Department of Pathogen Biology, College of Basic Medical Science, Jilin University, Changchun 130021, China; jianghl23@mails.jlu.edu.cn (H.J.); zhaoxueli1008@163.com (X.Z.); chwang24@mails.jlu.edu.cn (C.W.); hyshi@jlu.edu.cn (H.S.); ljh@jlu.edu.cn (J.L.); zhaocy@jlu.edu.cn (C.Z.)

**Keywords:** *Enterococcus faecalis*, phage biofilm, root canal, periapical periodontitis

## Abstract

*Enterococcus faecalis* (*E. faecalis*) is a major pathogen responsible for refractory apical periodontitis (RAP). It can penetrate deep into dentinal tubules, form persistent biofilms, and exhibit antibiotic resistance, thereby limiting the efficacy of conventional antimicrobial treatments. Bacteriophages (phages), due to their strong lytic activity and host specificity, have emerged as promising alternatives. In this study, a novel strictly lytic phage, ZXL-01, was isolated from lake water in Jilin, China. ZXL-01 demonstrated remarkable stability under extreme conditions, including thermal tolerance at 60 °C for 1 h and a wide pH range (4–11). Whole-genome sequencing (GenBank accession number: ON113334) revealed a genome of 40,804 bp with no virulence or tRNA genes, confirming its identity as an *E. faecalis* phage. Importantly, ZXL-01 exhibited potent antibiofilm activity, reducing biofilm biomass by approximately 69.4% in the inhibition group and 68.4% in the lysis group (both *p* < 0.001). In an in vitro root canal infection model induced by *E. faecalis*, scanning electron microscope (SEM) observations confirmed that ZXL-01 effectively inhibited biofilm formation and disrupted mature biofilms. These findings highlight the potential of ZXL-01 as a novel antimicrobial agent for the treatment of *E. faecalis*-associated apical periodontitis.

## 1. Introduction

Periapical periodontitis is mainly caused by a mixture of anaerobic microorganisms and their toxic products in the root canal system acting on periapical tissues through the apical foramen [1]. Endodontic therapy is currently the preferred treatment for periapical periodontitis. However, the incomplete removal of the infection source from the root canal can result in the progression from periapical periodontitis to refractory periapical periodontitis (RAP) [2,3]. *Enterococcus faecalis* (*E. faecalis*) is a common commensal bacterium in the digestive tract and oral cavity of humans and animals, has a prevalence of 33% in the dental pulp of RAP patients, and is considered one of the major pathogens of RAP [4,5]. *E. faecalis* has a strong penetration ability, can invade into the deeper layers of dentinal tubules, and forms a biofilm that provides a diffusion barrier, preventing antimicrobial agents from penetrating the biofilm. Bacteria within the biofilm can also maintain a low metabolic rate, reducing their susceptibility to antibiotics [6]. Not only that, but the long-term unregulated clinical use of antibiotics has led to a growing problem of resistance in bacteria [7]. Various studies conducted globally have shown an increasing rate of acquired cases of vancomycin-resistant enterococcal strains, with resistant strains to linezolid, tetracycline, and daptomycin antibiotics also being identified [8,9,10,11]. Calcium hydroxide solution, sodium hypochlorite solution, and chlorhexidine solution, which are currently used in clinical practice, can only inhibit *E. faecalis* and do not completely kill the bacteria within the biofilm [12,13,14]. Therefore, it is crucial to develop a new weapon against this bacterium.

Bacteriophages (phages) are the most abundant and ubiquitous organisms on Earth [15]. As viruses, they specialize in hunting bacteria. Their effective bactericidal ability and host specificity make them well-suited for use in the treatment of bacterial infections. Unfortunately, since the 1940s, when antibiotics were discovered and widely used, phages have only been used as a molecular and genetic research tool, without being developed towards clinical therapeutics. In recent years, with the misuse of antibiotics and the emergence of an increasing number of multidrug-resistant bacteria, people have realized that they should not continue to rely exclusively on antibiotics and gradually regained interest in phage therapy [16,17]. In this study, a new lytic phage, ZXL-01, was isolated from lake waters in Jilin, China, and its morphological, biological, and genetic properties were investigated. In addition, we constructed an in vitro model of *E. faecalis* root canal infection using an isolated tooth as carrier, explored phage therapy for periapical periodontitis caused by *E. faecalis*, and evaluated the effect of ZXL-01 on the cleavage of mature biofilm in the root canal of the tooth under scanning electron microscopy (SEM).

## 2. Materials and Methods

### 2.1. Materials

#### 2.1.1. Bacterial Strains and Growth Conditions

A total of 30 *E. faecalis* strains were obtained from bacterial stocks preserved in our laboratory. These isolates were identified utilizing the automated microorganism identification system (VITEK^®^ 2 Compact, bioMérieux SA, Marcy-l’Étoile, France) and were subsequently designated as HZB-01 through HZB-30. Additionally, the resistance profiles of these strains to eight antibiotics—tigecycline (TGC), ampicillin (AMP), penicillin G (PEN-G), linezolid (LZD), vancomycin (VAN), levofloxacin (LVX), high-level gentamicin (HLG), and erythromycin (ERY)—were determined using the aforementioned system. The culturing of host bacteria was performed on brain–heart infusion (BHI) agar and BHI broth at 37 °C, followed by preservation at −80 °C in 20% (*v*/*v*) glycerol [18].

#### 2.1.2. Reagents and Instruments

Brain–heart infusion (BHI) agar and BHI broth were obtained from Qingdao Hope Bio-Technology Co., Ltd. (Qingdao, China). Suspension Medium (SM) buffer was purchased from Wuhan Karnos Science and Technology Co., Ltd. (Wuhan, China). DNase I (1 mg/mL) and RNase A (1 mg/mL) were purchased from Takara Biomedical Technology Co., Ltd. (Beijing, China). Chloroform was purchased from Beijing Dingguo Changsheng Biotechnology Co., Ltd. (Beijing, China). The transmission electron microscope (TEM, JEM-1200EX) used in this study was from Japan Electronics and Optics Laboratory (Tokyo, Japan). The #10 to # 20 root canal file (K-file) and G-drill (1#) used in this study were from Dongguan Linsky Medical Device Technology Co., Ltd. (Dongguan, China). The scanning electron microscope (SEM, JSM-7900F) used in this study was from Japan Electronics and Optics Laboratory (Tokyo, Japan).

### 2.2. Methods

#### 2.2.1. Isolation and Purification of ZXL-01

Lake water samples were collected from a lake in Jilin, China, followed by the addition of 1.8 g CaCl_2_ (0.6% *w*/*v*) to the 300 mL water sample. The mixture was then subjected to centrifugation at 5000 rpm for 10 min, and the resulting supernatant was retained. Three flasks were designated as 1, 2, and 3 and were each inoculated with 100 mL of the supernatant and 100 mL of BHI broth. Flask 1 was inoculated with strains HZB-01 to HZB-10, flask 2 with strains HZB-11 to HZB-20, and flask 3 with strains HZB-21 to HZB-30. Subsequent incubation was carried out at 37 °C for 12 h, followed by filtration through a 0.22 μm filter (Millex^®^-GP, Merck KGaA, Darmstadt, Germany). A volume of 500 μL of each *E. faecalis* strain (HZB-01 to HZB-30) was mixed with 5.5 mL of diluted BHI agar (final concentration: 2.6% *w*/*v*), which had been cooled to approximately 50 °C. The mixture was immediately poured into a sterile centrifuge tube and then spread evenly onto a BHI agar plate. After the agar solidified, 10 μL of phage filtrate from flask No. 1 was spotted onto each of the plates containing strains HZB-01 to HZB-10. Similarly, 10 μL of filtrate from flask No. 2 was spotted onto plates with strains HZB-11 to HZB-20, and filtrate from flask No. 3 was applied to plates with strains HZB-21 to HZB-30. After standing at room temperature for 5 min, all plates were incubated at 37 °C for 12 h to allow the formation of visible plaque-forming units (PFUs) on the bacterial lawns [19].

The well-formed PFUs were then isolated and introduced into *E. faecalis* strains, followed by an incubation period of 12 h at 37 °C. The mixture was once again filtered (0.22-μm Millex^®^-GP), and then 100 μL of diluted filtrate, 100 μL of *E. faecalis*, and 5.5 mL of diluted BHI agar (final concentration of 2.6% *w*/*v* BHI agar) which was cooled to about 50 °C were mixed in a centrifuge tube. We quickly poured the mixture onto a BHI agar plate, and after the agar solidified, the plates were incubated at 37 °C (double-layer method). After obvious PFUs appeared in the bacterial lawn, the above purification process was repeated until homogeneity in PFU size and morphology was achieved [20].

#### 2.2.2. Transmission Electron Microscope (TEM)

The methodology for ZXL-01 concentration and purification was adapted from previously established protocols, with slight modifications [21]. In brief, large-scale cultures of ZXL-01 were prepared and treated with DNase I (1 mg/mL) and RNase A (1 mg/mL) at final concentrations of 1 μg/mL. The resulting mixture was then subjected to a series of treatments, including the addition of NaCl and polyethylene glycol (PEG) 8000, followed by centrifugation and resuspension in SM buffer. This was followed by the addition of chloroform, with the upper liquid phase preserved after centrifugation. A sample of this phase and glutaraldehyde aldehyde (0.5% *w*/*v*) were placed on carbon-coated copper grids, stained with phosphotungstic acid (PTA, 2% *w*/*v*), and observed under a TEM at an accelerating voltage of 80 kV.

#### 2.2.3. Titer Assay and Host Range Determination of ZXL-01

The phage titer was determined by using the double agar overlay plaque assay. We mixed 100 μL of serially diluted phage solution with 100 μL of *E. faecalis* and then added 5 mL of diluted BHI agar which was cooled to about 50 °C, poured the mixture onto a BHI agar plate, and incubated it at 37 °C for 12 h. After obvious PFUs appeared in the bacterial lawn, we could then deduce the titer of the phage stock solution based on the multiple of dilutions and the total number of PFUs on the bacterial law [22].

To determine the host range of ZXL-01, we resuscitated and cultured *E. faecalis* strains HZB-01 to HZB-30 preserved in our laboratory to the logarithmic phase and mixed 500 μL of each bacterial solution with the 5.5 mL of diluted BHI agar which was cooled to about 50 °C. After the agar solidified, we dripped 10 μL of ZXL-01 onto each of the plates. After waiting for five minutes, the plates were incubated at 37 °C for 12 h, and we could then determine whether ZXL-01 could infect the bacteria based on whether or not PFUs are present on the bacterial law.

#### 2.2.4. Optimal Multiplicity of Infection (MOI) and One-Step Growth Curve Determination of ZXL-01

In order to determine the optimal MOI of ZXL-01, we added 100 μL of *E. faecalis* (2 × 10^6^ CFU/mL) to each of the 6 flasks and 100 μL of already-diluted ZXL-01 solution of different titers so that the ratio of the titer of the ZXL-01 solution to the concentration of *E. faecalis* in the 6 flasks was 100, 10, 1, 0.1, 0.01, and 0.001. After incubation at 37 °C for 12 h, the mixture was filtered (0.22-μm Millex^®^-GP) to obtain the solution of the zygotic ZXL-01. Afterwards, the double agar overlay plaque assay was performed. The MOI corresponding to the experimental group with the highest ZXL-01 titer was the optimal MOI for ZXL-01.

A total of 500 μL of ZXL-01 was mixed with 500 μL of *E. faecalis* at an MOI of 0.1 and adsorbed at 37 °C for 10 min. The supernatant was removed after centrifugation at 10,000 rpm for 30 s and the precipitate was retained. The precipitate was resuspended with 1 mL of BHI broth and the mixture was again centrifuged at 10,000 rpm for 30 s. The supernatant was removed and the precipitate was retained. We repeated this process twice, and the final precipitate was resuspended in 20 mL of BHI broth; the mix was incubated at 37 °C. During 30 min after the start of incubation, samples were taken at 5 min intervals, and after 30 min, samples were taken at 10 min intervals. The double agar overlay plaque assay was used to determine the titer of ZXL-01 in each sample to plot the one-step growth curve [23].

#### 2.2.5. Stability of ZXL-01 at Different Temperatures and pH

In order to evaluate the thermal stability of ZXL-01, we incubated the ZXL-01 preparation (1 × 10^9^ PFU/mL) in a water bath of 50 °C, 60 °C, 70 °C, and 80 °C, sampled it at 10 min intervals, and measured its titer using the double agar overlay plaque assay. Similarly, in order to evaluate the pH stability of ZXL-01, a ZXL-01 preparation (1 × 10^9^ PFU/mL) was added to the BHI broth with a pH of 3, 4, 5, 6, 7, 8, 9, 10, 11, and 12; the titer was measured by the double agar overlay plaque assay after incubation at 37 °C for 1 h.

#### 2.2.6. Sequencing and Bioinformatics Analysis of Genome

Phenol–chloroform extraction methods were used to purify ZXL-01 DNA. In brief, 600 μL of the purified ZXL-01 particles were treated with 2.5 µL DNase I (1 mg/mL) and 0.5 µL RNase A (1 mg/mL), and the mixture was incubated for 1 h at 37 °C. Then, 20 μL of sodium dodecyl sulfate (SDS, 10% *w*/*v*), 2.5 μL of protease K (10 mg/mL), and 20 μL of ethylenediaminetetraacetic acid (EDTA, pH 8) were added to the mixture and incubated for 1 h at 56 °C. An equal volume of tris-saturated phenol was added to the cooled mixture; after centrifugation at 4 °C and 12,000 rpm for 10 min, the supernatant was transferred to a centrifuge tube containing an equal volume of phenol–chloroform–isoamyl alcohol (25:24:1) and centrifuged at 4 °C and 12,000 rpm for 5 min. Then, an equal volume of chloroform was added to the supernatant, and it was centrifuged at 4 °C and 12,000 rpm for 15 min. A total of 200 µL of sodium acetate and 4 mL of anhydrous ethanol were added to 2 mL of the supernatant and stored at 20 °C. After 30 min, the mixture was centrifuged at 4 °C and 12,000 rpm for 20 min, and the DNA pellet was washed with 75% ethanol, then air-dried at room temperature, and resuspended in 50 µL of deionized water [24].

Whole-genome sequencing was performed by Lc-bio Technologies (Hangzhou) Co., Ltd. (Hangzhou, China). The tRNAscan-SE website was used to detect the presence of tRNAs in the ZXL-01 genome. CG view was used to map the circular genes. The VFDB database was used to predict whether ZXL-01 contains virulence genes. The open reading frames (ORFs) of ZXL-01 were predicted using ORFs Finder, the function of each open reading frame of ZXL-01 was predicted and annotated using BLAST function on the NCBI website (https://www.ncbi.nlm.nih.gov/). And the genome annotation map of ZXL-01 was drawn using Prokee website (https://proksee.ca/). In order to study the relationship between ZXL-01 and other members of the *Siphoviridae* family, the whole-genome sequence of ZXL-01 and the protein sequence of the terminase large subnit were subjected to BLAST in NCBI. The phylogenetic tree of ZXL-01 was constructed using MEGA11 software, and the evolutionary relationship was analyzed using the tree [25].

#### 2.2.7. Effect of ZXL-01 on Bacterial Biofilm

To evaluate the inhibitory and removal effects of phage ZXL-01 on *E. faecalis* biofilms, two assays were conducted.

For the inhibition assay, 1 mL of *E. faecalis* suspension and 1 mL of BHI broth were added to each well of a 12-well plate. ZXL-01 was added at a multiplicity of infection (MOI) of 0.1, while no phage was added in the control group. The cultures were incubated at 37 °C for 36 h. Biofilm formation was assessed by crystal violet staining, and biofilm biomass was quantified by measuring absorbance at OD_570_.

For the biofilm removal assay, *E. faecalis* was first cultured in BHI broth (1 mL each) for 36 h at 37 °C to allow biofilm formation. Then, ZXL-01 was added at an MOI of 0.1, and the plates were incubated for an additional 8 h. The remaining biofilms were stained with crystal violet, and the lytic effect of ZXL-01 on mature biofilms was evaluated by measuring the OD_570_ value [26].

#### 2.2.8. Effect of ZXL-01 on In Vitro Model of Periapical Periodontitis

A total of 6 teeth voluntarily relinquished by extraction patients were collected from a dental hospital in Changchun. The isolated teeth had intact root canals, no caries damage, and the complete development of the periapical region. The root canals were explored with a #10 to # 20 root canal file (K-file) and the working length was established to be 1 mm shorter than the apical foramen. The pulp was removed from the root canal and the root canal opening was widened with a G-drill (1#). The isolated tooth was placed upright in a 10 mL centrifuge tube containing 1.5 mL of BHI broth. After autoclaving at 120 °C for 30 min and then incubating at 37 °C for 48 h, if the medium in the centrifuge tube remained clear after 48 h, it proved that the sterilization effect was good enough to carry out the next experiment. The prepared teeth were divided into an experimental group and a control group with three isolated teeth in each group. For each isolated tooth, 50 μL of *E. faecalis* was added to the root canal, and 4.5 mL of BHI broth was added to the centrifuge tube to completely submerge the isolated teeth. The prepared teeth were incubated at 37 °C and the BHI broth was renewed every 24 h [27]. After 21 days, root canals were searched under SEM at 40× after the control isolated teeth were split, and biofilms on the surface of the root canals were scanned and photographed at 2000×, 5000×, and 10,000×. The BHI broth in the experimental group was replaced and 50 μL of ZXL-01 was added to the isolated root canals of the teeth, incubated at 37 °C, and the procedure was renewed every 24 h. After 5 days, the biofilm condition of *E. faecalis* in the root canals was photographed by SEM.

#### 2.2.9. Statistical Analysis

SPSS version 13.0 (SPSS, Inc., Chicago, IL, USA) was used for all statistical analyses. Differences with *p* < 0.05 were considered statistically significant. Error bars indicate standard deviations.

## 3. Results

### 3.1. Antimicrobial Resistance of E. faecalis

Antimicrobial susceptibility testing of 30 *E. faecalis* strains showed that all strains were susceptible to TGC, AMP, PEN-G, and VAN. LZD resistance was detected in one strain (HZB-12), and two strains (HZB-15 and HZB-20) exhibited intermediate susceptibility. LVX resistance remained high, with 17 isolates (56.7%) classified as resistant. In addition, resistance to HLG was observed in 15 isolates (50.0%), while ERY resistance was the most prevalent, affecting 28 out of 30 strains (93.3%).

Notably, 16 isolates exhibited multidrug resistance (MDR), defined as non-susceptibility to two or more antimicrobial agents. Among them, several isolates—such as HZB-03, HZB-13, HZB-14, HZB-16, HZB-20, HZB-25, and HZB-26—exhibited resistance to three or more antibiotics, including linezolid, levofloxacin, high-level gentamicin, and erythromycin (Table 1).

### 3.2. Isolation and Morphology of ZXL-01

**Figure 1:**
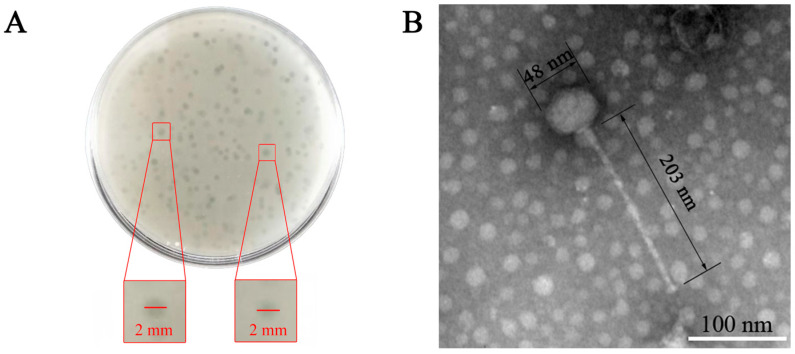
Morphological of ZXL-01. (**A**) Morphology of ZXL-01 plaques. (**B**) Morphology of ZXL-01 observed by transmission electron microscopy (TEM).

A bacteriophage specific to *E.* *faecalis* was isolated from untreated lake water in Jilin, China, and subsequently purified, and ZXL-01 was denominated. The ZXL-01 elicited the formation of clear plaques, approximately 1–2 mm in diameter, upon a bacterial lawn of the host *E. faecalis* (Figure 1A). TEM analysis revealed that ZXL-01 possesses a head diameter approximating 48 nm and a tail length of about 203 nm (Figure 1B). According to the classification criteria of the International Committee on Taxonomy of Viruses (ICTV), bacteriophage ZXL-01 is classified within the family *Siphoviridae* of the order *Caudovirales*.

### 3.3. Host Range Analysis

The host range of ZXL-01 was determined based on its ability to form plaques on a panel of 30 clinical *E. faecalis* isolates. Phage ZXL-01 exhibited lytic activity against 15 of the tested strains, whereas no plaque formation was observed on the remaining 15 isolates (Table 2).

### 3.4. Biological Characteristics of ZXL-01

**Figure 2:**
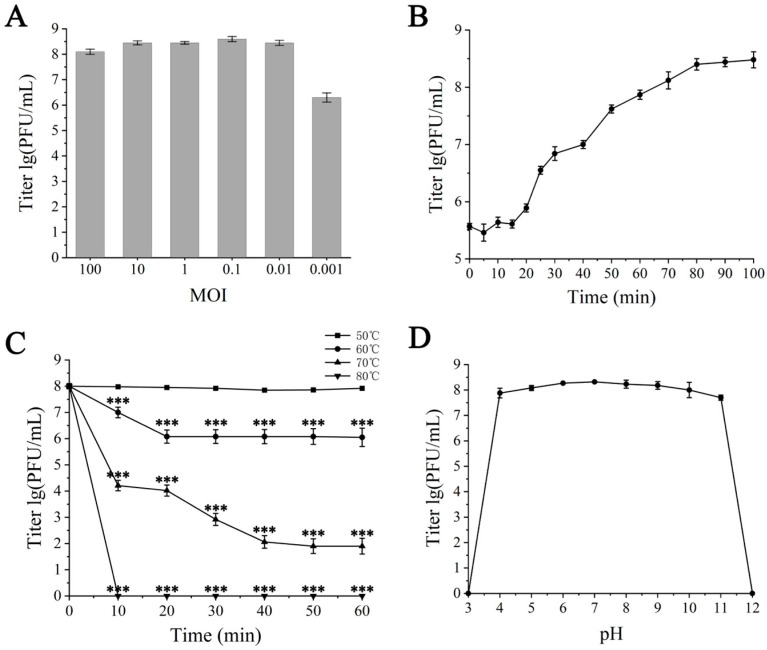
Biological characterization of ZXL-01. (**A**) Optimal multiplicity of infection (MOI) determination. (**B**) One-step growth curve. (**C**) Stability of ZXL-01 at different temperatures (comparison of phage activity at the same time at 50 °C; *** *p* < 0.001). (**D**) Stability of ZXL-01 at different pH.

At an MOI of 0.1, the maximum titer of the progeny phages was obtained, which was 4 × 10^8^ PFU/mL, thereby establishing the optimal MOI for ZXL-01’s interaction with *E. faecalis* as 0.1 (Figure 2A). One-step growth curve analysis conducted at an MOI of 0.1 unveiled a 20 min latent period and a 70 min burst period, with a burst size of approximately 30 PFU/cell after 90 min (Figure 2B). Thermal stability assessments revealed that ZXL-01 maintained high activity levels post-incubation at 60 °C, although activity notably diminished at 70 °C, with no detectable viral particles remaining after a 10 min incubation at 80 °C (Figure 2C). Moreover, pH stability assays showed ZXL-01 to be stable across a pH range of 4 to 11, beyond which its stability was compromised (Figure 2D).

### 3.5. Analysis of ZXL-01 Genome

**Figure 3:**
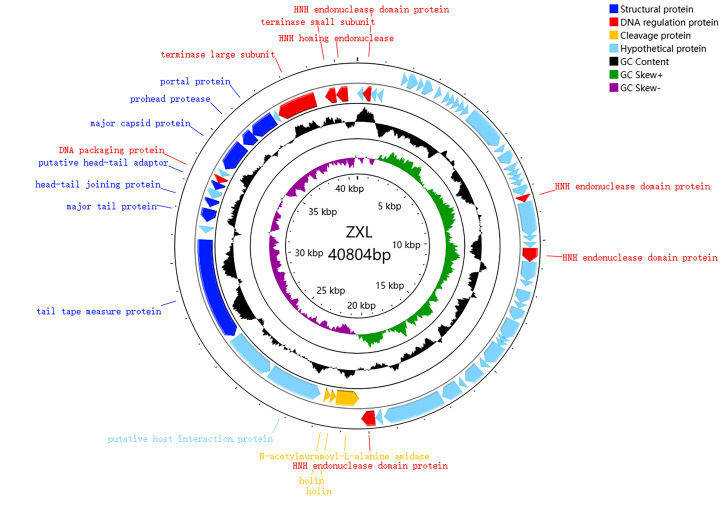
Genomic annotation map of phage ZXL-01, with genes represented by arrows. The functional ORFs are categorized into four groups: structural proteins (dark blue), DNA regulation proteins (red), cleavage proteins (yellow), and hypothetical proteins (light blue). GC content is shown in black, with outward bars indicating regions with GC content higher than the genome average, and inward bars indicating lower GC content. GC skew is shown in green and purple (calculated as [G − C]/[G + C]); outward indicates a value greater than 0, and inward indicates a value less than 0.

The complete genome of ZXL-01 was sequenced, elucidating a DNA composition spanning 40,804 base pairs, with a GC content of 35%. Notably, the genome has no tRNA and no virulence gene. The whole-genome sequence has been deposited in the NCBI GenBank database (https://www.ncbi.nlm.nih.gov/nuccore, accessed on 24 March 2023) under the accession number ON113334. Annotation revealed 63 putative protein-coding genes [open reading frames (ORFs)], with 39 ORFs being positive-sense and 24 negative-sense (Table 3). Among these, 19 ORFs were annotated with putative functions, while 44 ORFs remain with unknown functions. The 19 ORFs with known functions could be categorically divided into three modules, namely structural proteins, DNA regulation proteins, and cleavage proteins (Table 4, Figure 3).

**Figure 4:**
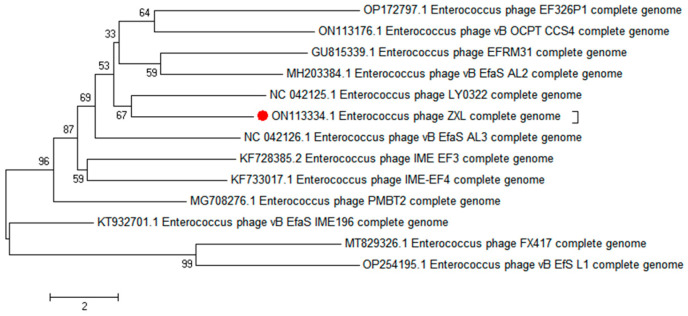
Phylogenetic analysis of phage ZXL-01 (ZXL-01 has been marked in red).

**Figure 5:**
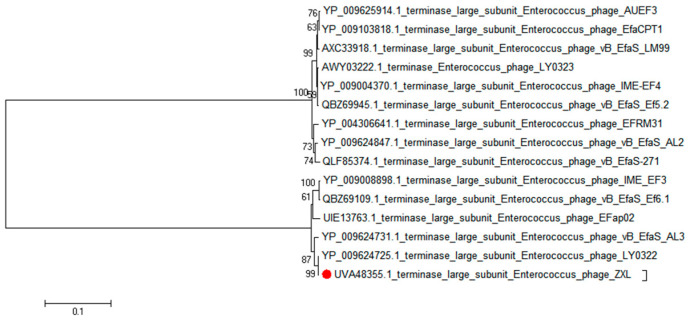
Phylogenetic analysis of the phage ZXL-01 terminase large subnit (ZXL-01 has been marked in red).

A phylogenetic analysis was conducted to elucidate the genetic relatedness of ZXL-01 to other phages. Comparative genomic analysis, based on both the complete genome sequence and the terminase large subunit protein, revealed that phage ZXL-01 is closely related to *E. faecalis* phage LY0322 (NC_042125), sharing 99.99% of their nucleotide identity as determined by BLASTn analysis (Figure 4 and Figure 5). Notably, the DNA packaging, structural, and cleavage-related proteins of ZXL-01 and LY0322 exhibit highly conserved amino acid sequences, suggesting a shared evolutionary origin and a potential overlap in host range. These findings provide valuable insights for the future development of phage cocktails targeting polymicrobial infections.

### 3.6. Phage Treatment on Bacterial Biofilm

**Figure 6:**
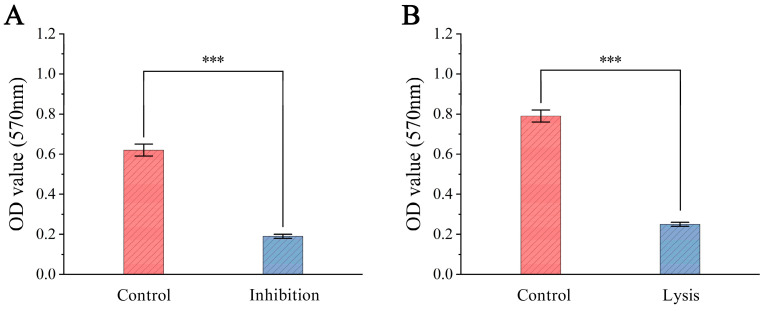
(**A**) Inhibition of biofilm formation by phage ZXL-01. (**B**) Lysis of pre-formed biofilms by phage ZXL-01 (comparison of phage activity at the same time at 50 °C; *** *p* < 0.001).

To evaluate the inhibitory and lytic effects of ZXL-01 on *E. faecalis* biofilms, biofilm biomass was measured based on average OD_570_ values. After 48 h of incubation, the phage-treated group showed an approximately 69.4% reduction in biofilm formation compared to the control group, with OD_570_ decreasing from about 0.62 to 0.19 (Figure 6A). Additionally, when 1 mL of ZXL-01 (PFU/mL) was applied to 36 h mature biofilms and incubated for another 8 h at 37 °C, the biomass decreased by approximately 68.4%, with OD_570_ dropping from around 0.79 to 0.25 (Figure 6B). These findings indicate that ZXL-01 exhibits potent activity in both preventing the formation and promoting the lysis of *E. faecalis* biofilms.

**Figure 7:**
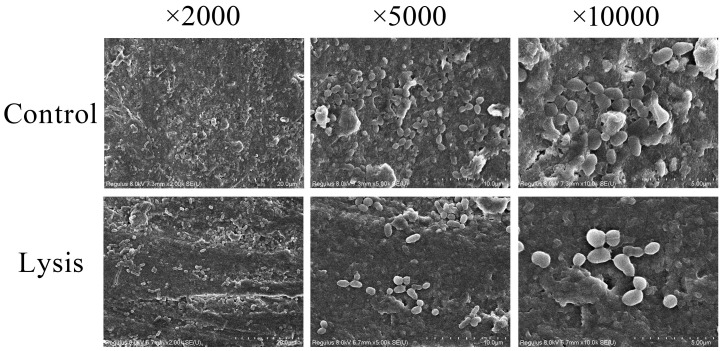
ZXL-01 inhibits *E. faecalis* biofilm formation in root canals.

To further assess its application potential, ZXL-01 was used to lyse biofilm in periapical periodontitis caused by *E. faecalis*. A total of 50 μL of *E. faecalis* was inoculated into the root canal of an isolated tooth, followed by submersion in BHI broth medium and incubation at 37 °C for 21 days. SEM analysis at this juncture revealed the successful establishment of an *E. faecalis* root canal infection model. Subsequent to a 5-day ZXL-01 intervention in the root canal, SEM observations highlighted ZXL-01’s capacity to effectively lyse the biofilm within the root canal, thereby affirming the potential applicability of ZXL-01 in mitigating biofilm growth within the root canal in cases of periapical periodontitis caused by *E. faecalis* (Figure 7).

## 4. Discussion

*E. faecalis* is the main conditional causative agent of nosocomial infections and forms a dense biofilm in the root canal, which may lead to treatment failure in periapical periodontitis due to the incomplete removal of the biofilm and cause periapical periodontitis to evolve into RAP [6,28]. Because of the complex structure of root canals, mechanical methods of removing infected nerves accompanied by the use of antimicrobial rinsing solutions are clinically ineffective in completely removing *E. faecalis* biofilm from root canals, and the long-term use of antimicrobial rinsing solutions can make the bacteria in the lesion no longer sensitive to the rinse, which makes the treatment of periapical periodontitis very difficult. There is an urgent need to develop new methods capable of removing the biofilm. However, the phage’s host-specific infection and relatively narrow cleavage spectrum are among the barriers to its further application. To overcome these limitations, strategies such as screening more lytic phages, combining phages with antibiotics, or administering phage cocktails should be investigated [29]. It has been reported that combinations of multiple phages, combinations of phages and antibiotics, or combinations of phages with other anti-microbial agents can extend the host range and thus improve the effectiveness of phage application [30]. Therefore, it is important to enrich the phage library by isolating new phages that are more applicable to clinical therapy.

In this study, a novel lytic *E. faecalis* phage, ZXL-01, was isolated and characterized using 30 clinical *E. faecalis* isolates as host strains. These isolates were obtained from hospitalized patients and tested for antibiotic susceptibility using agents that are routinely employed in clinical antimicrobial susceptibility testing. The antimicrobial susceptibility results revealed that all strains were susceptible to TGC, AMP, PEN-G, and VAN. However, resistance to LVX and HLG was observed in 56.7% and 50.0% of the isolates, respectively, and 93.3% were resistant to ERY. LZD resistance was rare, with one fully resistant strain and two showing intermediate susceptibility. Overall, the isolates displayed diverse resistance profiles, including multidrug resistance.

ZXL-01 exhibited lytic activity against 15 of the 30 clinical isolates. Furthermore, one-step growth curve analysis showed that ZXL-01 has a latent period of approximately 20 min, a lysis period of 70 min, and an average burst size of 30 PFU per cell (Figure 2B). The environmental tolerance of phage affects the efficacy of phage therapy. The protein shell of the phage surface is susceptible to denaturation under high-temperature, acidic, or alkaline environments, leading to phage inactivation. ZXL-01 can withstand temperatures as high as 60 °C for 1 h, with substantial inactivation occurring only when the temperature reaches 70 °C (Figure 2C). Regarding the stability of ZXL-01 at different pH, ZXL-01 has a wide pH tolerance range and can maintain stable activity in the pH range of 4–11. (Figure 2D).

The genome of ZXL-01 was sequenced and deposited in the GenBank database under the accession number ON113334. It consists of 40,804 base pairs with a GC content of 35% and contains no tRNA genes or virulence factors. A total of 63 open reading frames (ORFs) were predicted, of which 39 are on the positive strand and 24 on the negative strand. These ORFs are closely arranged and compactly organized. Nineteen ORFs with known functions were categorized into three functional modules: structural protein, DNA regulation, and lysis. In the DNA regulation module, the HNH homing endonuclease is involved in the mobility of genetic elements, while DNA packaging proteins, including the large and small subunits of terminase, play key roles in translocating DNA into empty capsids. Within the structural module, the major capsid protein—typical of tailed phages—forms an icosahedral head structure, and the prohead protease assists in head maturation. The head–tail joining proteins link the portal to tail components, while the tail tape measure protein determines tail length and forms the DNA injection channel. Multiple major tail proteins constitute the long, non-contractile tail of the phage. The lysis module includes N-acetylmuramoyl-L-alanine amidase, an enzyme that degrades the bacterial cell wall, facilitating DNA entry and bacterial lysis. Phylogenetic analysis based on the whole-genome and terminase large subunit amino acid sequences revealed that ZXL-01 is closely related to *Enterococcus* phage LY0322 (NC_042125), which shares a similar genome length (40,804 vs. 40,934 bp), GC content (35% vs. 34.8%), and number of ORFs (63 vs. 64). ZXL-01 also exhibits high sequence similarity with LY0322 in DNA packaging, structural, and lysis proteins. These similarities suggest that ZXL-01 and LY0322 may infect the same host species. Such insights into genomic and functional characteristics of ZXL-01 can aid in host prediction and support the rational design of phage combinations for treating polymicrobial infections.

Biofilm is an organized community of microorganisms regulated by signaling molecules, presenting a reticulated, three-dimensional structure consisting of the extracellular matrix of bacteria and containing a large number of conduits in its structure, which are keys to signaling pathways and energy exchange metabolism. Biofilm can act as a diffusion barrier, thus preventing the penetration of antimicrobials into the biofilm. Within a biofilm, bacteria can maintain a lower metabolic rate, which reduces susceptibility to antibiotics. *E. faecalis* can survive in a low metabolic state in a nutrient-poor root canal, and when the nutrient environment improves with the cessation of medication, it generates a new bacterial biofilm that can lead to re-infection [31]. In this experiment, to investigate the inhibition of *E. faecalis* biofilm formation by ZXL-01 and the lysis of formed *E. faecalis* biofilm, it was found that ZXL-01 exhibited potent antibiofilm activity, reducing biofilm biomass by approximately 69.4% in the inhibition group and 68.4% in the lysis group (both *p* < 0.001). In order to further explore its potential application in endodontic therapy, an in vitro model of *E. faecalis* root canal infection was successfully constructed in this study. SEM observations confirmed that ZXL-01 was able to effectively cleavage the biofilm from the root canals after 5 days of incubation in the root canals.

In previous studies, phage vB_ZEFP, isolated from hospital wastewater in Egypt, exhibited a shorter latent period (10 min) and a higher burst size (110 PFU/cell), and both phages effectively disrupted biofilms in root canal models [32]. Phage PEf771 demonstrated complete lysis within 3 h, with an optimal MOI of 1, and outperformed ten commonly used antibiotics. Its genome is much larger (151,052 bp), encoding 197 proteins and 26 tRNAs, and showed robust protective effects in both in vitro and in vivo models, including rat models of periapical and intraperitoneal infections [33]. Additionally, two phages, vB_Efa29212_2e and vB_Efa29212_3e, targeting *E. faecalis* ATCC 29212, exhibited good thermal stability (4–50 °C) and resistance to disinfectants such as EDTA, sodium hypochlorite, and chlorhexidine. In a 21-day ex vivo bovine tooth model, they achieved a 54.6% reduction in biofilm, confirming their therapeutic potential [34].

In summary, ZXL-01 showed lytic activity against over half of the tested isolates. It has a latent period of 20 min, has a burst size of 30 PFU/cell, and maintains stability at 60 °C for 1 h and at a pH of 4–11. Its genome is 40,804 bp with a 35% GC content, lacking virulence and tRNA genes. Genomic analysis reveals close relation to *Enterococcus* phage LY0322. ZXL-01 effectively inhibits biofilm formation and disrupts established biofilms, demonstrating strong potential for clinical application in in vitro models of periapical periodontitis. Future studies should explore its therapeutic efficacy and safety in vivo to advance phage therapy for refractory periapical periodontitis.

## 5. Conclusions

In conclusion, this study describes the isolation, characterization, and application of a new lytic *E. faecalis* phage, ZXL-01. It belongs to the long-tailed phage family of the order Arbuscular mycobacteria and has no tRNA, no virulence genes, 63 ORFs, and 19 ORFs with predictable functions. The genome of ZXL-01 is currently sequenced and deposited in the GenBank database under the accession number ON113334. It has a short incubation period (20 min) and a wide pH range (pH 4–11) and temperature tolerance (60 °C). In this study, ZXL-01 significantly inhibited *E. faecalis* biofilm formation and cleaved already-formed biofilms within an in vitro model of periapical periodontitis induced by *E. faecalis*, suggesting that it has great potential for clinical use in the treatment of periapical periodontitis.

## Figures and Tables

**Table 1:** Antimicrobial resistance analysis of 30 *E. faecalis* strains.

No.	TGC	AMP	PEN-G	LZD	VAN	LVX	HLG	ERY
HZB-01	S	S	S	S	S	S	S	R
HZB-02	S	S	S	S	S	S	R	R
HZB-03	S	S	S	S	S	R	R	R
HZB-04	S	S	S	S	S	S	S	R
HZB-05	S	S	S	S	S	R	S	R
HZB-06	S	S	S	S	S	S	S	R
HZB-07	S	S	S	S	S	R	S	I
HZB-08	S	S	S	S	S	S	S	R
HZB-09	S	S	S	S	S	S	S	R
HZB-10	S	S	S	S	S	S	R	R
HZB-11	S	S	S	S	S	S	S	R
HZB-12	S	S	S	R	S	R	S	I
HZB-13	S	S	S	S	S	R	R	R
HZB-14	S	S	S	S	S	R	R	R
HZB-15	S	S	S	I	S	S	R	R
HZB-16	S	S	S	S	S	R	R	R
HZB-17	S	S	S	S	S	S	R	R
HZB-18	S	S	S	S	S	R	S	R
HZB-19	S	S	S	S	S	R	S	R
HZB-20	S	S	S	I	S	R	R	R
HZB-21	S	S	S	S	S	S	R	R
HZB-22	S	S	S	S	S	S	S	R
HZB-23	S	S	S	S	S	R	S	I
HZB-24	S	S	S	S	S	S	R	I
HZB-25	S	S	S	S	S	R	R	R
HZB-26	S	S	S	S	S	R	R	R
HZB-27	S	S	S	S	S	R	S	R
HZB-28	S	S	S	S	S	S	R	R
HZB-29	S	S	S	R	S	R	S	I
HZB-30	S	S	S	S	S	S	R	S

Three sensitivities of ZXL-01 to antimicrobial agents: insensitive (R), sensitive (S), and intermediary (I).

**Table 2:** Host range determination of ZXL-01.

No.	Plaque Formation	No.	Plaque Formation	No.	Plaque Formation
HZB-01	+	HZB-11	+	HZB-21	+
HZB-02	+	HZB-12	+	HZB-22	−
HZB-03	+	HZB-13	+	HZB-23	−
HZB-04	+	HZB-14	−	HZB-24	+
HZB-05	−	HZB-15	−	HZB-25	+
HZB-06	−	HZB-16	−	HZB-26	−
HZB-07	−	HZB-17	−	HZB-27	−
HZB-08	−	HZB-18	+	HZB-28	−
HZB-09	+	HZB-19	−	HZB-29	+
HZB-10	+	HZB-20	−	HZB-30	+

Two lysis categories of ZXL-01 to *E. faecalis*: clear lysis zone (+) and no lysis zone (−).

**Table 3:** ZXL-01 gene annotations.

CDS	Function	Strand	Start (bp)	End (bp)	Accession No.
1	hypothetical protein	−	25	204	UVA48295.1
2	HNH endonuclease domain protein	−	205	576	NC_042125.1
3	hypothetical protein	−	576	779	YP_009624666.1
4	hypothetical protein	−	862	1053	YP_009624667.1
5	hypothetical protein	+	1717	1929	YP_009624668.1
6	hypothetical protein	+	1942	2337	YP_009624669.1
7	hypothetical protein	+	2315	2563	YP_009624670.1
8	hypothetical protein	+	2567	2944	YP_009624671.1
9	hypothetical protein	+	3100	3294	YP_009624672.1
10	hypothetical protein	+	3475	3633	YP_009624674.1
11	hypothetical protein	+	3645	3866	YP_009624675.1
12	hypothetical protein	+	3859	4077	YP_009624676.1
13	hypothetical protein	+	4074	4313	YP_009624677.1
14	hypothetical protein	+	4310	4507	YP_009624678.1
15	hypothetical protein	+	4602	6188	YP_009624679.1
16	hypothetical protein	+	6277	6465	YP_009624680.1
17	hypothetical protein	+	6538	6999	YP_009624681.1
18	hypothetical protein	+	7048	7320	YP_009624682.1
19	hypothetical protein	+	7322	7483	YP_009624683.1
20	hypothetical protein	+	7485	7694	YP_009624684.1
21	hypothetical protein	+	7697	7888	YP_009624685.1
22	hypothetical protein	+	7914	8288	YP_009624686.1
23	HNH endonuclease domain protein	+	8281	8544	YP_009624687.1
24	hypothetical protein	+	8534	9832	QOI67902.1
25	hypothetical protein	+	9829	10,005	YP_009624689.1
26	hypothetical protein	+	10,065	10,259	YP_009624690.1
27	HNH endonuclease domain protein	+	10,273	10,806	AWY03188.1
28	hypothetical protein	+	10,787	11,530	YP_009624692.1
29	hypothetical protein	+	11,523	11,729	YP_009624693.1
30	hypothetical protein	+	11,886	12,392	AXC33931.1
31	hypothetical protein	+	12,373	12,600	QOI67895.1
32	hypothetical protein	+	12,597	13,076	YP_009624696.1
33	hypothetical protein	+	13,087	13,866	QOI67893.1
34	hypothetical protein	+	13,839	14,000	YP_009624820.1
35	hypothetical protein	+	13,993	14,175	YP_009624699.1
36	hypothetical protein	+	14,165	14,983	YP_009624700.1
37	hypothetical protein	+	14,984	15,238	YP_009624701.1
38	hypothetical protein	+	15,316	16,023	YP_009624702.1
39	hypothetical protein	+	16,094	16,318	YP_009624703.1
40	hypothetical protein	+	16,353	17,081	QOI67887.1
41	hypothetical protein	+	17,117	19,408	QOI67886.1
42	hypothetical protein	+	19,472	19,741	YP_009624706.1
43	HNH endonuclease domain protein	+	19,744	20,274	YP_009624707.1
44	N-acetylmuramoyl-L-alanine amidase	−	20,316	21,311	BCU01267.1
45	holin	−	21,314	21,547	YP_009624709.1
46	holin	−	21,562	21,807	YP_009624710.1
47	putative host interaction protein	−	21,986	24,340	QOI67880.1
48	hypothetical protein	−	24,352	26,433	WAX15190.1
49	tail tape measure protein	−	26,508	30,878	AXC33914.1
50	hypothetical protein	−	31,135	31,446	QBZ69822.1
51	major tail protein	−	31,626	32,192	YP_004306651.1
52	head–tail joining protein	−	32,271	32,636	AXC33940.1
53	hypothetical protein	−	33,040	32,633	YP_009613291.1
54	putative head–tail adaptor	−	33,037	33,372	AXC33942.1
55	DNA packaging protein	−	33,344	33,646	BCU01280.1
56	hypothetical protein	−	33,684	33,884	AXC33965.1
57	major capsid protein	−	34,014	35,257	AWY03218.1
58	prohead protease	−	35,327	35,914	AWY03219.1
59	portal protein	−	35,877	37,028	YP_009624723.1
60	hypothetical protein	−	37,033	37,221	YP_004306642.1
61	terminase large subunit	−	37,267	38,988	YP_009624725.1
62	terminase small subunit	−	39,409	39,882	YP_009624726.1
63	HNH homing endonucleases	−	39,883	40,395	QOI67925.1

Strand direction of the gene: positive strand (+) and negative strand (−).

**Table 4:** Predicted ORFs and functions of ZXL-01.

Function	Feature ID
structural protein module	tail tape measure protein (ORF49), major tail protein (ORF51), head–tail joining protein (ORF52), head–tail adaptor (ORF54), major capsid protein (ORF57), prohead protease (ORF58), portal protein (ORF59)
DNA regulation protein module	HNH homing endonuclease (ORF2, ORF23, ORF27, ORF43, ORF63), DNA packaging protein (ORF55), terminase large subnit (ORF61), terminase small subnit (ORF62)
cleavage protein module	N-acetylmuramoyl-L-alanine amidase (ORF44), holin (ORF45, ORF46)

## Data Availability

The complete genome sequence of phage ZXL-01 was deposited in GenBank under an accession number [ON113334]. The deposited data are publicly available as of the date of publication.
